# Development of a machine learning-based radiomics model of perivascular adipose tissue for predicting stroke risk in patients with asymptomatic carotid stenosis: a multicenter study

**DOI:** 10.3389/fradi.2025.1738298

**Published:** 2026-01-21

**Authors:** Jinhong Sun, Cheng Ma, Guihan Lin, Weiyue Chen, Weiming Hu, Zhuohang Shi, Ting Zhao, Jie Zhang, Jianhua Wu, Xiongying Yi, Hua Yang, Suhong Ye, Lei Xu, Yongjun Chen, Weiqian Chen

**Affiliations:** 1 Department of Vascular Surgery, Lishui Central Hospital, The Fifth Affiliated Hospital of Wenzhou Medical University, Lishui, China; 2 Zhejiang Key Laboratory of Imaging and Interventional Medicine, The Fifth Affiliated Hospital of Wenzhou Medical University, Lishui, China; 3 Department of Radiology, Lishui Central Hospital, The Fifth Affiliated Hospital of Wenzhou Medical University, Lishui, China; 4 Department of Radiology, The Second Affiliated Hospital of Wenzhou Medical University, Wenzhou, China; 5 Department of Radiology, Lishui People’s Hospital, Lishui, China

**Keywords:** asymptomatic carotid stenosis, machine learning, perivascular adipose tissue, radiomics, stroke

## Abstract

**Background:**

Our work aims to develop and evaluate a combined model that integrates clinical features, conventional computed tomography angiography (CTA) features, and radiomics features of perivascular adipose tissue (PVAT) to identify asymptomatic carotid stenosis (ACS) patients at high risk for short-term stroke.

**Methods:**

We enrolled 582 ACS patients confirmed by CTA from three medical centers and divided them into a training set (*n* = 188), an internal validation set (*n* = 85), and two independent external validation sets (set 1, *n* = 157; set 2, *n* = 152). Radiomics features of PVAT were extracted from CTA images, and dimensionality reduction was performed to identify predictive features. Five machine learning classifiers were employed to construct radiomics models, and the model with the highest AUC was selected to generate the radiomics score (Rad-score). Clinical factors associated with stroke were determined using univariate and multivariate logistic regression analyses to construct a clinical model. A combined model integrating clinical factors and the Rad-score was subsequently developed, and a nomogram was created to provide a visual tool for stroke risk prediction. We assessed model performance comprehensively through calibration curves, discrimination analysis, reclassification, and clinical application.

**Results:**

A total of nine optimal radiomics features were ultimately selected from the CTA images. Across the four datasets, the AUC values of the five classifier models ranged from 0.643 to 0.869, 0.716 to 0.826, 0.651 to 0.858, and 0.638 to 0.848, respectively, with the XGBoost model demonstrating the best performance. The combined model, incorporating hypertension, soft plaque, and the Rad-score as variables, achieved AUCs of 0.911, 0.868, 0.882, and 0.871, respectively, across the four datasets.

**Conclusions:**

A combined model based on PVAT imaging features around carotid plaques can effectively predict the short-term stroke risk in ACS patients. This model may be expected to provide an important auxiliary tool for clinical prognosis assessment and treatment decisions, with potential clinical application value.

## Introduction

Ischemic stroke serves as an important reason for patients' death and disability, with harmful effects that cannot be overlooked (1). Approximately 25%–30% of ischemic stroke cases are closely associated with carotid artery atherosclerosis (CAS) (2, 3). CAS can be categorized into symptomatic carotid stenosis (SCS) and asymptomatic carotid stenosis (ACS). SCS patients typically exhibit more pronounced clinical manifestations and a greater risk of stroke, often prompting clinical attention and timely surgical intervention (4, 5). Conversely, ACS presents insidiously with a lack of overt clinical symptoms and is characterized only by imaging findings; however, the associated stroke risk is equally concerning. Current international guidelines for the management of ACS patients lack a consensus (6, 7), leading to delayed or inappropriate clinical interventions for some high-risk ACS patients, ultimately resulting in stroke (8). Therefore, early identification and intervention for ACS patients are crucial for stroke prevention.

Computed tomography angiography (CTA) can be used to evaluate stenosis and assist in risk stratification through plaque characteristics (9). Currently, CAS patients' stroke risk assessment relies primarily on vascular stenosis degree, although correlation is not absolute (10). Perivascular adipose tissue (PVAT), which surrounds the carotid artery, is recognized as an endocrine organ able to secrete bioactive substances. Under inflammatory conditions, PVAT releases proinflammatory factors that can exacerbate intraplaque inflammation, thereby increasing plaque instability (11). In recent years, accumulating evidence has suggested that CTA is capable of precisely detecting and measuring PVAT inflammatory alterations linked to atherosclerosis (12) and ischemic episodes (13). For example, researchers have utilized CTA to assess perivascular fat density (PFD) in carotid arteries, revealing an association between increased PFD and both unstable atherosclerotic plaques and an elevated risk of cerebrovascular events. Zhang et al. (14) evaluated PFD around carotid plaques in 206 patients and reported an important relationship between PFD and cerebrovascular events, such as intraplaque hemorrhage. These findings suggest that PVAT may serve as a crucial predictor, effectively characterizing carotid plaque instability. However, the inflammatory state is a complex biological process, and relying solely on PFD to reflect the inflammatory status of PVAT is insufficient.

As an innovative approach, radiomics facilitates the efficient extraction of numerous quantifiable characteristics from clinical imaging data. This technique converts digital medical images into high-dimensional datasets (15), offering extensive information that surpasses conventional visual interpretation. Recently, radiomics-based approaches have been widely utilized in tumor staging, early diagnosis, differentiation, prognosis prediction, and treatment evaluation (16, 17) technology also offers novel avenues for atherosclerosis research (18, 19). To date, PVAT-based radiomics has demonstrated superior performance in coronary arteries, enabling effective prediction of cardiovascular events. Huang et al. (20) evaluated the utility of features in predicting adverse cardiovascular events via pericoronary adipose tissue (PCAT)-based ML algorithms, and the results indicated that the inclusion of PVAT radiomics features dramatically enhanced clinical models' performance. A multicenter research by You et al. (21) compared radiomics models' performance of PCAT and epicardial adipose tissue in predicting major adverse cardiovascular events (MACE) within 3 years and concluded that the PCAT-based model outperformed the EAT-based model. Recently, researchers have focused on developing models using the imaging features of carotid plaques to forecast CAS patients' stroke risk. However, the predictive value of PVAT radiomics features for ACS patients' stroke risk remains uncertain.

Our work aimed to establish a prediction model by integrating imaging features of PVAT around carotid plaques to forecast short-term stroke risk in ACS patients. Our study is designed to provide clinical guidance for therapeutic decision-making in ACS patients, thereby improving stroke prevention.

## Methods

### Patient selection and clinical data

**Figure 1:**
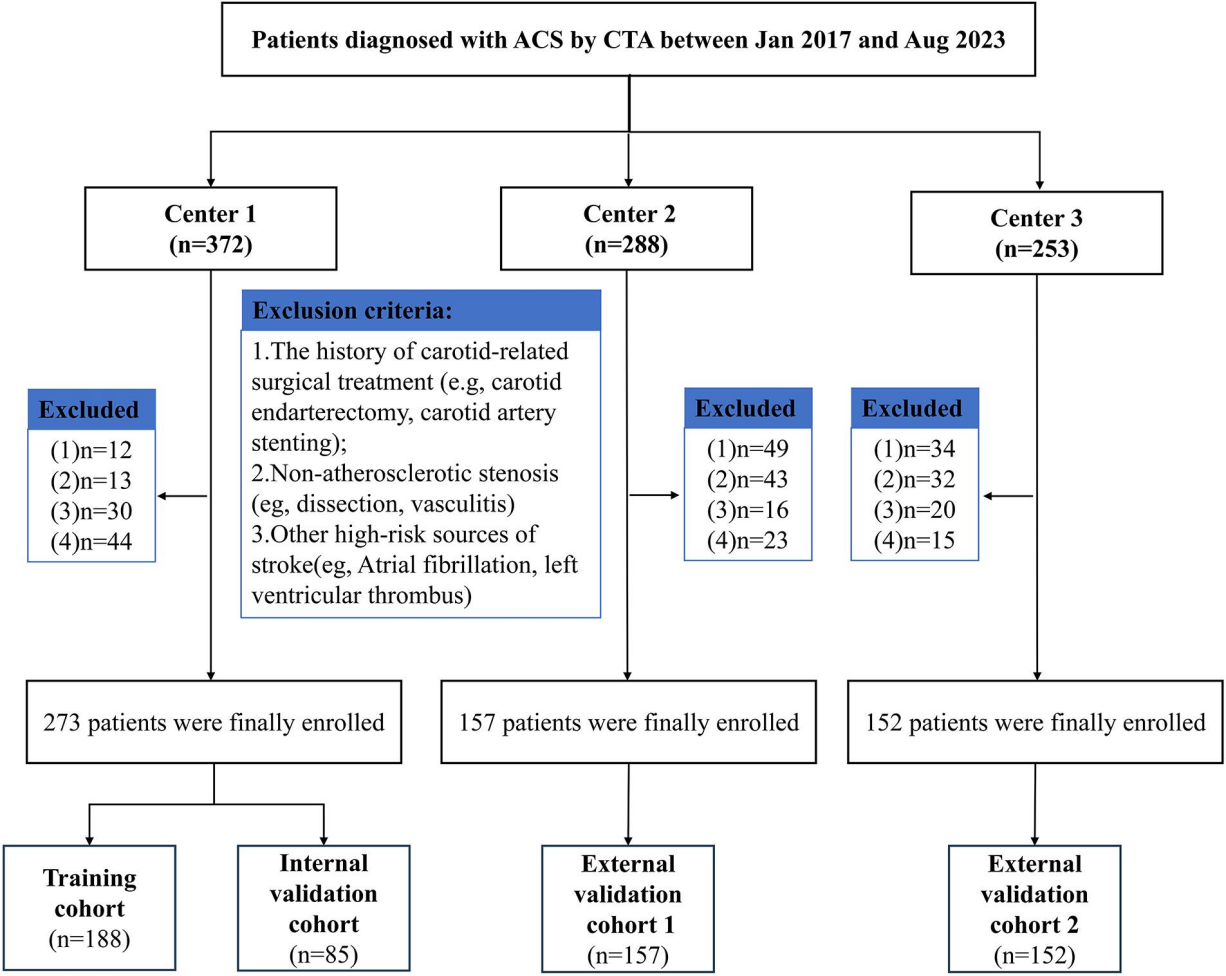
Recruitment pathway for eligible patients in our work.

Our work was approved by the Institutional Review Boards of the Fifth Affiliated Hospital of Wenzhou Medical University (Center 1), Second Affiliated Hospital of Wenzhou Medical University (Center 2), and Sixth Affiliated Hospital of Wenzhou Medical University (Center 3). (no. 2025[I]-169-02). As this was a retrospective study, informed consent from patients was not required. We collected data from patients diagnosed with ACS by CTA examination at Centers 1, 2, and 3 between January 2017 and August 2023. ACS is commonly defined as the presence of atherosclerotic narrowing of the proximal internal carotid artery by ≥50% at the level of bifurcation in individuals with no history of recent (within the last 6 months) ischemic stroke/transient ischemic attack (TIA) involving the ipsilateral carotid territory (22). The enrollment criteria were as follows: (1) patients diagnosed with ACS based on CTA; (2) carotid CTA images of sufficient quality for radiomics analysis; and (3) at least 2 years of complete follow-up data from the date of diagnosis, with clear documentation of stroke events during follow-up. Because all patients had at least 2 years of follow-up, the predictive models developed in this study were designed to estimate short-term stroke occurrence. We adopted exclusion criteria as follows: (1) prior carotid revascularization (carotid endarterectomy, carotid stenting, etc.); (2) carotid stenosis due to non-atherosclerotic causes, such as arteritis, dissection, or radiation injury; and (3) the presence of other high-risk factors for stroke (like atrial fibrillation or left ventricular thrombus). Stroke events during follow-up were defined in Supplementary Appendix E1. The detailed patient enrollment process refers to Figure 1.

### CTA imaging protocol and image analysis

Head and neck CTA examinations were conducted using multi-detector CT scanners (Somatom Force, Siemens; Somatom Definition Flash, Siemens; Aquilion One, Toshiba). The patient was placed in a supine position with head extended and instructed to maintain normal respiration. The scan direction was from the feet to the head, encompassing the region from the sternal angle to the vertex of the skull. Acquisition parameters of such protocols are provided in Supplementary Table S1.

### Segmentation of plaque PVAT

**Figure 2:**
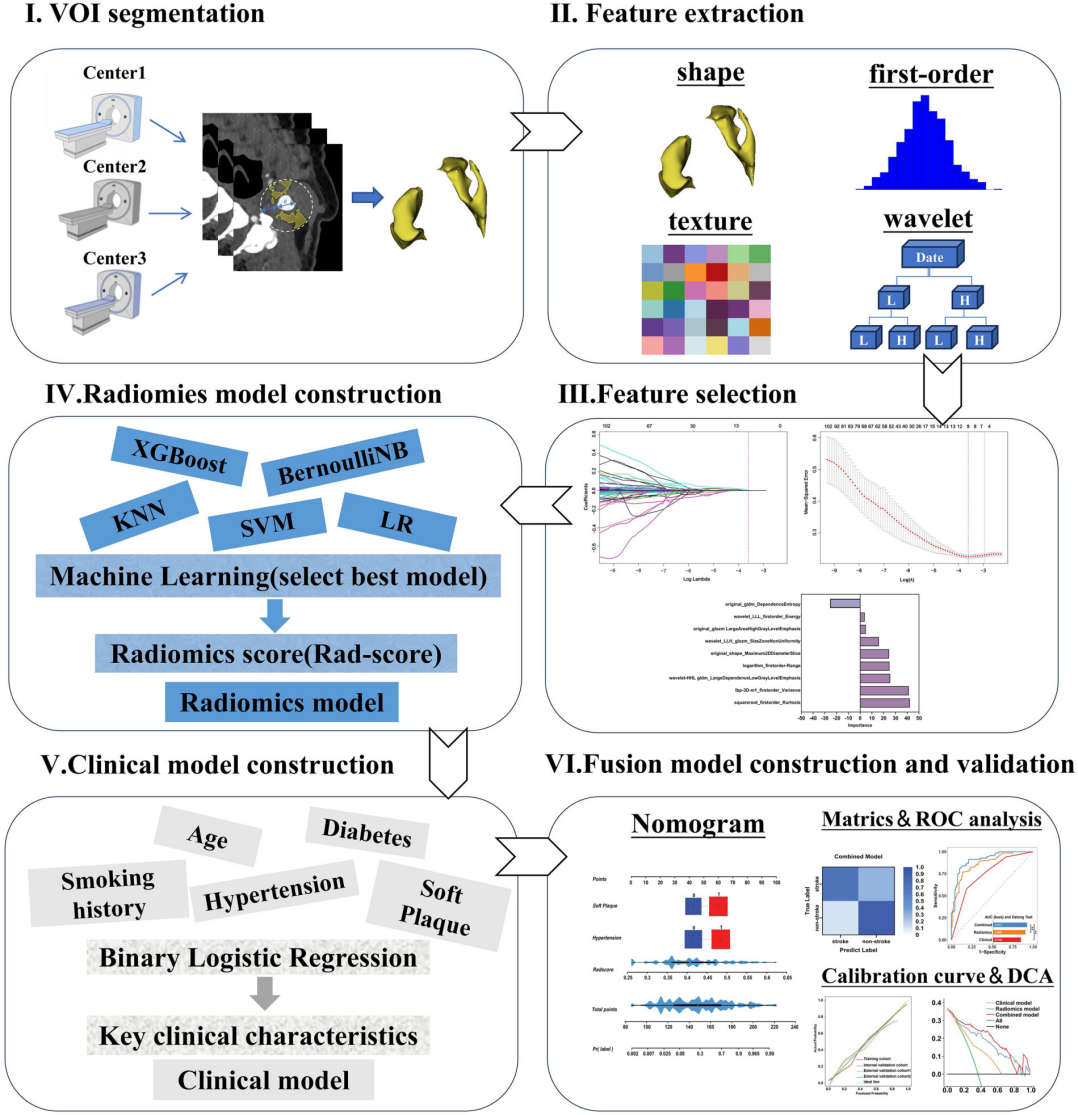
Radiomics workflow of this study.

**Figure 3:**
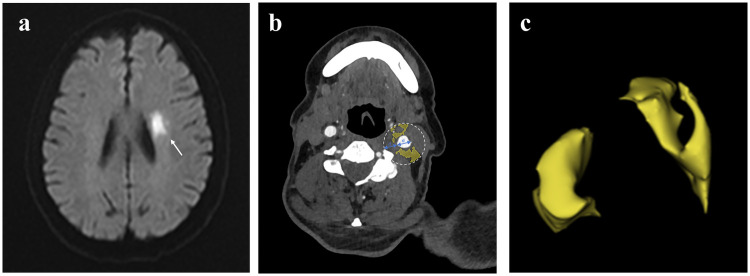
Segmentation of PVAT on CTA. **(a)** A case that experienced a stroke; **(b)** PVAT (yellow) segmented automatically by the segmentation software; and **(c)** an image showing the three-dimensional PVAT region (yellow).

The radiomics workflow of this study is illustrated in Figure 2. Two radiologists, each with over 10 years of experience in diagnostic radiology, performed the segmentation of the PVAT volume of interest (VOI) using dedicated radiomics software (3D Slicer). Any disagreements encountered during the segmentation process were resolved through discussion and consensus between the two radiologists. The methodology was as follows: the measurement range extended 2 cm cephalad and caudad from the carotid bifurcation (total length, 4 cm). We defined PVAT as adipose tissue within radial distance from the outer vessel wall equal to the carotid artery diameter (CT value range, −190 to −30 HU). The software semiautomatically segmented the adipose tissue based on these parameters, and the resulting VOI was used for subsequent radiomics analysis. Representative images from ACS patients are shown in Figure 3.

### Radiomics feature extraction

Following image preprocessing (Supplementary Appendix E2), we employed the open-source Python package “pyradiomics” to extract radiomics features from each ROI. We extracted seven radiomics feature classes, namely, glcm, shape, glszm, gldm, glrlm, ngtdm, and first order. To ensure consistency of extracted features, we utilized the R package irr to perform intra- and interobserver intraclass correlation coefficient (ICC) analysis to assess feature reproducibility, retaining stable features (ICC >0.8) for subsequent statistical analysis (Supplementary Appendix E3). To ensure feature scaling consistency across different samples, we performed *Z*-score normalization (Supplementary Appendix E4) on radiomics features. A three-step procedure including variance thresholding, K-best univariate selection, and least absolute shrinkage and selection operator (LASSO) regression was subsequently executed (Supplementary Appendix E5).

### Radiomics model construction

The radiomics features selected via LASSO regression were subsequently input into the following machine learning (ML) classifiers to build a radiomics model: k-nearest neighbors (KNN), support vector machine (SVM), logistic regression (LR), Bernoulli naive Bayes (BernoulliNB), and eXtreme Gradient Boosting (XGBoost). Rationale and considerations for selecting these five ML classifiers are detailed in Supplementary Appendix E6. Hyperparameter combinations for each classifier were optimized automatically by grid search, implemented in Python (Supplementary Appendix E7). Furthermore, five classifiers were validated using a validation cohort. We assessed the predictive performance of radiomics feature models via the area under the receiver operating characteristic (ROC) curve (AUC), sensitivity, specificity, and precision. We chose a classifier with the highest average AUC value in the validation cohort as the optimal classifier, which was then utilized to sort key radiomics features of ACS patients, calculating a radiomics score (Rad-score) to reflect the relative stroke risk of ACS patients to construct a radiomics model. For validating its diagnostic performance, we analyzed the Rad-score distribution between the stroke and non-stroke groups.

### Development and validation of predictive models

Associations between clinical and conventional CTA features and the occurrence of stroke in ACS patients were assessed utilizing univariate LR analysis. Clinical predictors with *p* < 0.05 were incorporated into a multivariate LR analysis to build a clinical model. Multivariate analysis and backward stepwise regression, which are based on the Akaike information criterion, were subsequently conducted to build a combined model and corresponding nomogram in the training cohort, combining Rad-score and significant clinical predictors. To enhance the interpretability of the combined model, we performed a visualization analysis of the three most important radiomics features. We assessed collinearity and excluded variables with a variance inflation factor (VIF) exceeding 10 and *p* > 0.05. These models were then validated using a validation cohort. We assessed models' prediction performance via ROC analysis, calibration curve analysis, and decision curve analysis (DCA). Based on the reference standard of follow-up outcomes, we determined percentages of true positives, false positives, true negatives, and false negatives via ROC analysis and presented their results in a confusion matrix. Bootstrapped calibration curves, with 1,000 resamples, were generated. We conducted DCA for visualizing the net benefit of clinical decisions and utilized net reclassification improvement (NRI) and integrated discrimination improvement (IDI) values for quantifying clinical utility and the net benefit of different models.

### Statistical analysis

We conducted data analysis utilizing SPSS 25.0, MedCalc 22.014, and R 4.4.3, and assessed data normality via the Kolmogorov–Smirnov test. We denoted continuous variables as means ± standard deviations (SDs) or medians and interquartile ranges, whereas we reported categorical variables as counts and percentages. For comparisons, continuous variables were analyzed using Student's *t*-tests or Mann–Whitney *U* tests, whereas categorical variables were analyzed using *χ*^2^ tests or Fisher's exact tests. Univariate LR analysis was employed to evaluate associations between ACS patients’ clinical characteristics and stroke occurrence; features with *p* < 0.05 were enrolled in multivariate LR analysis for further investigation. Furthermore, several R packages were utilized, including “glmnet” for LASSO regression, “rms” for LR analysis and calibration curve plotting, “rmda” for DCA, and “PredictABEL” for calculating NRI and IDI. We conducted ROC analysis via MedCalc software and compared disparities in AUC values among different models via the DeLong test. All statistical tests were two-sided, and *p* < 0.05 was viewed to imply significance.

## Results

### Patient characteristics and clinical model construction

Table 1 and Supplementary Table S2 present a summary of the clinical and CTA characteristics for a total of 582 patients across training, internal validation, external validation 1, and external validation 2 cohorts. Overall, the four cohorts demonstrated balance and comparability. After univariate and multivariate regression analyzes, we identified hypertension [odds ratio (OR): 3.86, confidence interval (CI): 1.97–7.82, *p* < 0.001] and soft plaque (OR: 4.29, CI: 2.03–9.43, *p* < 0.001) as independent predictors of stroke in ACS patients and used them to build a clinical model (Supplementary Table S3). No collinearity was detected, as variance inflation factors (VIFs) for predictors were 1.063 and 1.121, respectively.

**Table 1:** Comparison of clinical and CTA features between stroke and non-stroke groups in patients with ACS.

Characteristics	Training cohort (*n* = 188)			Internal validation cohort (*n* = 85)			External validation cohort 1 (*n* = 157)			External validation cohort 2 (*n* = 152)		
	Stroke (*n* = 69)	Non-stroke (*n* = 119)	*p*	Stroke(*n* = 31)	Non-stroke (*n* = 54)	*p*	Stroke (*n* = 54)	Non-stroke (*n* = 103)	*p*	Stroke(*n* = 64)	Non-stroke (*n* = 88)	*p*
Age, years*	56.71 ± 11.04	56.34 ± 10.38	0.820	53.03 ± 10.58	57.39 ± 9.73	0.058	56.11 ± 9.96	58.67 ± 10.44	0.141	59.36 ± 12.00	56.78 ± 12.70	0.209
Sex (%)			0.113			0.356			0.871			0.665
Female	30 (43.48)	66 (55.46)		14 (45.16)	30 (55.56)		26 (48.15)	51 (49.51)		29 (45.31)	43 (48.86)	
Male	39 (56.52)	53 (44.54)		17 (54.84)	24 (44.44)		28 (51.85)	52 (50.49)		35 (54.69)	45 (51.14)	
BMI, kg/m^2^*	24.50 ± 1.08	24.45 ± 0.96	0.736	24.60 ± 0.96	24.51 ± 0.92	0.682	25.06 ± 2.62	24.33 ± 2.30	0.074	23.93 ± 2.38	24.39 ± 2.56	0.268
Smoking status (%)			0.141			0.607			0.483			0.368
No	39 (56.52)	54 (45.38)		19 (61.29)	30 (55.56)		32 (59.26)	55 (53.4)		36 (56.25)	43 (48.86)	
Yes	30 (43.48)	65 (54.62)		12 (38.71)	24 (44.44)		22 (40.74)	48 (46.6)		28 (43.75)	45 (51.14)	
Diabetes (%)			0.584			0.758			0.971			**0**.**035**
No	33 (47.83)	52 (43.7)		16 (51.61)	26 (48.15)		25 (46.3)	48 (46.6)		26 (40.62)	51 (57.95)	
Yes	36 (52.17)	67 (56.3)		15 (48.39)	28 (51.85)		29 (53.7)	55 (53.4)		38 (59.38)	37 (42.05)	
Hypertension (%)			**<0**.**001**			**0**.**004**			**0**.**002**			**0**.**009**
No	18 (26.09)	69 (57.98)		10 (32.26)	35 (64.81)		14 (25.93)	53 (51.46)		22 (34.38)	49 (55.68)	
Yes	51 (73.91)	50 (42.02)		21 (67.74)	19 (35.19)		40 (74.07)	50 (48.54)		42 (65.62)	39 (44.32)	
Hyperlipidemia (%)			0.051			0.886			0.242			0.579
No	38 (55.07)	48 (40.34)		15 (48.39)	27 (50)		23 (42.59)	54 (52.43)		32 (50.00)	48 (54.55)	
Yes	31 (44.93)	71 (59.66)		16 (51.61)	27 (50)		31 (57.41)	49 (47.57)		32 (50.00)	40 (45.45)	
CAD (%)			0.084			0.255			0.871			0.651
No	38 (55.07)	50 (42.02)		15 (48.39)	33 (61.11)		26 (48.15)	51 (49.51)		30 (46.88)	38 (43.18)	
Yes	31 (44.93)	69 (57.98)		16 (51.61)	21 (38.89)		28 (51.85)	52 (50.49)		34 (53.12)	50 (56.82)	
Family history (%)			0.968			0.296			0.962			0.986
No	34 (49.28)	59 (49.58)		18 (58.06)	25 (46.3)		28 (51.85)	53 (51.46)		29 (45.31)	40 (45.45)	
Yes	35 (50.72)	60 (50.42)		13 (41.94)	29 (53.7)		26 (48.15)	50 (48.54)		35 (54.69)	48 (54.55)	
Soft plaque (%)			**<0**.**001**			**<0**.**001**			**<0**.**001**			**0**.**027**
No	15 (21.74)	67 (56.3)		8 (25.81)	34 (62.96)		14 (25.93)	57 (55.34)		24 (37.5)	49 (55.68)	
Yes	54 (78.26)	52 (43.7)		23 (74.19)	20 (37.04)		40 (74.07)	46 (44.66)		40 (62.5)	39 (44.32)	
Ulcerated plaque (%)			**0**.**011**			0.104			0.254			0.175
No	42 (60.87)	93 (78.15)		19 (61.29)	42 (77.78)		29 (53.70)	65 (63.11)		44 (68.75)	51 (57.95)	
Yes	27 (39.13)	26 (21.85)		12 (38.71)	12 (22.22)		25 (46.30)	38 (36.89)		20 (31.25)	37 (42.05)	
Plaque thickness (mm)*	4.23 ± 1.40	4.22 ± 1.27	0.968	4.05 ± 1.49	4.32 ± 1.24	0.374	4.27 ± 1.15	3.93 ± 1.18	0.090	4.05 ± 1.41	4.13 ± 1.10	0.692
Plaque length (mm)*	14.70 ± 6.79	15.07 ± 5.42	0.683	15.83 ± 6.12	15.22 ± 6.68	0.673	14.67 ± 6.04	16.24 ± 5.10	0.089	15.85 ± 5.46	15.95 ± 5.62	0.912
TC (mmol/L)	3.53 (3.39,3.71)	3.51 (3.27,3.69)	0.615	3.42 (3.27,3.63)	3.49 (3.37,3.64)	0.302	3.50 (3.39,3.63)	3.47 (3.33,3.59)	0.256	3.46 (3.25, 3.76)	3.42 (3.20, 3.63)	0.349
TG (mmol/L)	1.18 (1.05,1.32)	1.19 (1.03,1.31)	0.895	1.10 (1.00,1.20)	1.12 (1.01,1.28)	0.482	1.13 (0.98,1.21)	1.14 (0.99,1.27)	0.489	1.20 (1.01, 1.32)	1.17 (1.03, 1.32)	0.536
HDL-C (mmol/L)	1.00 (0.93,1.09)	1.03 (0.93,1.11)	0.319	1.04 (0.93,1.10)	1.01 (0.95,1.16)	0.674	0.94 (0.84, 1.09)	1.02 (0.95, 1.10)	**0**.**002**	1.01 (0.94, 1.08)	0.99 (0.95, 1.06)	0.372
LDL-C (mmol/L)	1.79 (1.60,1.98)	1.88 (1.54,2.06)	0.410	1.91 (1.54,2.08)	1.90 (1.57,2.10)	0.816	1.94 (1.78,2.13)	1.90 (1.74,2.02)	0.096	1.85 (1.68, 2.03)	1.89 (1.77, 2.11)	0.082

ACS, asymptomatic carotid stenosis; CTA, computed tomography angiography; BMI, body mass index; CAD, coronary artery disease; TC, total cholesterol; TG, triglyceride; HDL-C, high-density lipoprotein cholesterol; LDL-C, low-density lipoprotein cholesterol.

Bold values indicate statistically significant differences (*p* < 0.05).

*Values expressed as mean ± standard deviation.

### Radiomics feature selection and signature construction

The intraobserver ICCs spanned from 0.834 to 0.939, whereas the interobserver ICCs spanned from 0.803 to 0.907. Feature selection results for the training cohort are provided in Supplementary Figure S1. Nine radiomics features were ultimately retained for predicting stroke occurrence in ACS patients via the variance threshold approach, K-best univariate selection, and LASSO regression algorithm, with the relative significance of the nine chosen features listed in Supplementary Figure S1C. Correlation heatmap implied that the chosen features derived from CTA were independent (Supplementary Figure S2). A radiomics signature was subsequently generated by combining all the selected features and employing five ML classifiers: KNN, SVM, LR, XGBoost, and BernoulliNB.

### Analysis of radiomics signature efficacy and model construction

**Figure 4:**
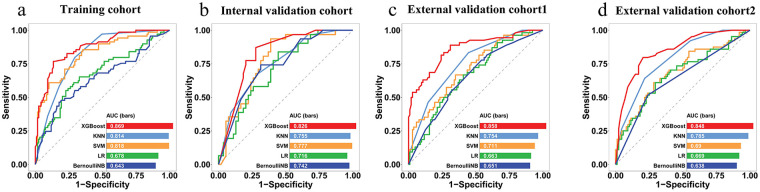
Receiver operating characteristic curves of the radiomics signatures with five ML classifiers in **(a)** training, **(b)** internal validation, **(c)** external validation cohort 1, and **(d)** external validation cohort 2.

Prediction performance of radiomics signature, which is based on five classifiers, is presented in Supplementary Figures S3a–c and Table 2 and includes training, internal validation, external validation 1, and external validation 2 cohorts. In comparative analysis, the XGBoost algorithm demonstrated superior performance, attaining peak average AUC (0.844) and accuracy (0.787) scores in three validation cohorts. Furthermore, the XGBoost classifier exhibited higher predictive performance than other classifiers within three validation cohorts, as evidenced by the ROC curves in Figure 4 and the DeLong test results in Supplementary Figures S3d–f. Accordingly, XGBoost was chosen as the preferred classification algorithm to compute Rad-scores for building a radiomics model. Ultimately, Rad-scores derived from XGBoost were significantly different between the non-stroke and stroke groups of ACS patients across all four datasets (all *p* < 0.001, Supplementary Figure S4).

**Table 2:** Diagnostic performance of varied ML-based radiomics signatures.

Model	Training cohort		Internal validation cohort		External validation cohort 1		External validation cohort 2	
	AUC (95%CI)	ACC (%)	AUC (95%CI)	ACC (%)	AUC (95%CI)	ACC (%)	AUC (95%CI)	ACC (%)
KNN	0.814 (0.754–0.874)	70.74	0.755 (0.652–0.858)	71.76	0.754 (0.681–0.827)	71.97	0.785 (0.711–0.859)	72.37
SVM	0.818 (0.752–0.884)	76.60	0.777 (0.672–0.882)	62.35	0.711 (0.629–0.793)	65.61	0.690 (0.614–0.766)	65.13
LR	0.678 (0.609–0.747)	67.55	0.716 (0.606–0.826)	67.05	0.662 (0.582–0.744)	64.96	0.669 (0.593–0.745)	64.47
XGBoost	0.869 (0.808–0.930)	77.66	0.826 (0.731–0.921)	77.64	0.858 (0.787–0.929)	80.89	0.848 (0.782–0.914)	77.63
BernoulliNB	0.643 (0.572–0.714)	64.36	0.716 (0.606–0.826)	69.41	0.651 (0.569–0.733)	63.69	0.638 (0.565–0.711)	65.79

AUC, area under the curve; ACC, accuracy; CI, confidence interval; KNN, k-nearest neighbors; SVM, support vector machine; LR, logistic regression; XGBoost, eXtreme Gradient Boosting; BernoulliNB, Bernoulli naive Bayes.

### Creation and verification of an individualized predictive nomogram

**Figure 5:**
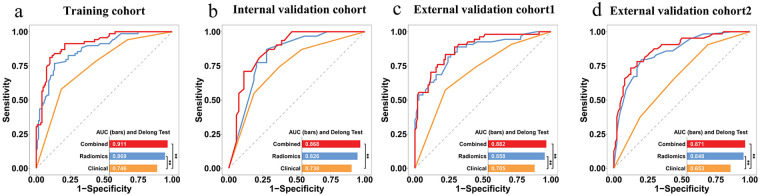
ROC curves for predicting stroke in ACS patients via different models. **(a)** training, **(b)** internal validation, **(c)** external validation cohort 1, and **(d)** external validation cohort 2. *indicates *p* < 0.05 by DeLong's test.

**Figure 6:**
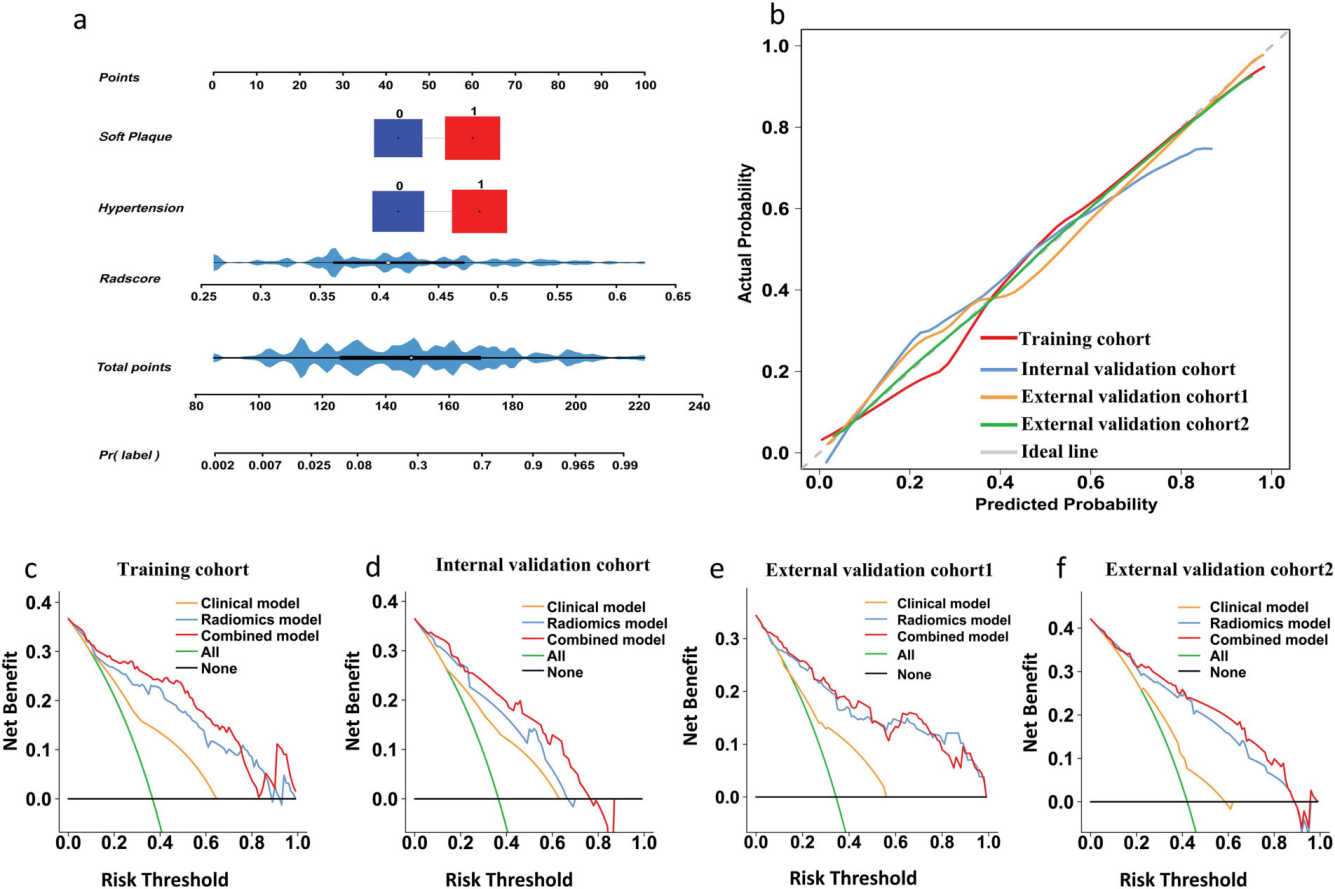
Nomogram, calibration curves, and decision curve analysis. **(a)** A combined nomogram combining rad-score based on XGBoost, hypertension, and ulcerated plaque for predicting the probability of stroke in ACS patients. **(b)** Calibration curves of combined model in training, internal validation, external validation cohort 1, and external validation cohort 2. **(c–f)** Decision curve analysis outcomes of clinical model, radiomics model, and combined model in four cohorts.

We built a combined predictive model by incorporating XGBoost-based Rad-scores with major clinical factors, including hypertension and soft plaques. Supplementary Figure S5 presents confusion matrices detailing the comparative performance of all three models across both training and three validation cohorts. The ROC curves for predicting stroke occurrence in ACS patients are presented in Figure 5. Incorporating Rad-scores significantly improved clinical model AUC values across training, internal validation, and external validation 1 and 2 cohorts, increasing from 0.746 to 0.911 (*Z* = 5.066, *p* < 0.001), from 0.738 to 0.868 (*Z* = 2.580, *p* = 0.010), from 0.705 to 0.882 (*Z* = 4.232, *p* < 0.001), and from 0.653 to 0.871 (*Z* = 4.952, *p* < 0.001), respectively, in Table 3. However, there were no significant differences in AUC values between the radiomics model and the combined model in internal, external validation 1, and external validation 2 cohorts (all *p* > 0.05). Ultimately, the combined model was visualized as a nomogram to individualize the prediction of stroke risk in ACS patients (Figure 6a). Calibration curves exhibited outstanding consistency between predicted probabilities of stroke and actual observations in ACS patients (Figure 6b). Furthermore, the DCA results indicated that the overall net benefit of the radiomics model and combined model was superior to that of the clinical model across the most reasonable threshold probabilities in four cohorts (Figures 6c–f). Moreover, including Rad-scores in the combined model realized 0.301 total NRI (95% CI: 0.162–0.441, *p* < 0.05) and 0.308 IDI (95% CI: 0.234–0.375, *p* < 0.05). We found similar outcomes in three validation cohorts (Supplementary Figure S6), demonstrating the improved predictive efficiency and classification accuracy of the combined model in predicting stroke occurrence in ACS patients. Nevertheless, comparative analysis revealed no huge disparities in either NRI or IDI when the radiomics model was compared with the combined model in all four cohorts (all *p* values >0.05). Supplementary Figure S7 shows the visualization of the first three features with the highest importance.

**Table 3:** Predictive performance of clinical model, radiomics model, and combined model in different cohorts.

Cohort	Model	AUC (95%CI)	SEN (%)	SPE (%)	ACC (%)
Training	Clinical	0.746 (0.678–0.807)	57.97	81.51	72.87
	Radiomics	0.869 (0.812–0.913)	76.81	86.55	82.98
	Combined	0.911 (0.861–0.947)	81.16	89.92	86.70
Internal validation	Clinical	0.738 (0.631–0.827)	74.19	62.96	67.06
	Radiomics	0.826 (0.728–0.900)	87.10	72.22	77.65
	Combined	0.868 (0.777–0.931)	70.97	88.89	82.35
External validation 1	Clinical	0.705 (0.627–0.775)	57.41	77.67	70.70
	Radiomics	0.858 (0.794–0.909)	88.89	68.93	75.80
	Combined	0.882 (0.821–0.928)	83.33	77.67	79.62
External validation 2	Clinical	0.653 (0.571–0.728)	90.62	30.68	55.92
	Radiomics	0.848 (0.781–0.901)	79.69	79.55	79.61
	Combined	0.871 (0.808–0.920)	78.12	82.95	80.92

AUC, area under the curve; ACC, accuracy; CI, confidence interval; SEN, sensitivity; SPE, specificity.

## Discussion

Our work developed a combined model based on clinical features, conventional plaque CTA features, and radiomics features of PVAT to identify ACS patients at high risk of short-term stroke. The combined model demonstrated good discrimination ability in the training set (AUC = 0.911), internal validation set (AUC = 0.868), external validation set 1 (AUC = 0.882), and external validation set 2 (AUC = 0.871). This combined model exhibited apparently superior predictive ability than the clinical model (hypertension and soft plaques), highlighting the added value of radiomics in stroke risk stratification. This model can provide personalized treatment decisions for high-risk populations among ACS patients and reduce the medical burden.

The onset of ischemic stroke typically involves the interplay of multiple risk factors and pathophysiological mechanisms. In this study, univariate and multivariate LR analyses identified hypertension and soft plaques as independent risk factors for predicting stroke risk in ACS patients. The AUC values for the clinical models constructed based on these factors were 0.746, 0.738, 0.705, and 0.653, respectively. Elevated blood pressure is an established contributor to ischemic stroke risk, potentially because chronic high blood pressure damages vascular endothelial cells and promotes the formation of atherosclerotic plaques, which can lead to plaque rupture or thromboembolism, obstructing cerebral blood vessels and triggering acute cerebral infarction (23, 24). Among ACS patients, the stroke cohort demonstrated a significantly greater prevalence of soft plaques than non-stroke patients did. Soft plaque contains a lipid-rich necrotic core (LRNC) composed of accumulated lipids and inflammatory components. Persistent inflammation and enzymatic degradation weaken the fibrous cap, making the plaque vulnerable to rupture under hemodynamic stress. Once ruptured, the LRNC releases lipids and proinflammatory mediators that activate platelets and coagulation cascades, resulting in thrombus formation. The subsequent thromboembolism can occlude cerebral arteries, ultimately leading to ischemic stroke (7).

Recent advancements have been made in the use of PVAT radiomics features of the carotid artery to identify symptomatic plaques. Nie et al. (9) investigated 203 patients categorized into symptomatic and asymptomatic groups as per acute/subacute stroke or transient ischemic attack occurrence. Their radiomics feature model for identifying symptomatic plaques achieved 0.834 maximum AUC in the test set. However, their research was single-center, potentially leading to overfitting, and lacked an external validation dataset to assess the diagnostic efficacy of the ML model. In contrast, the PVAT radiomics feature model developed in our study demonstrated robust performance in predicting 2-year stroke events, with AUCs of 0.869, 0.826, 0.858, and 0.848 in the training, internal validation, external validation 1, and external validation 2 cohorts, further confirming the PVAT model's value in identifying stroke risk in ACS patients. Radiomics features in this study were generated by first screening a large number of extracted features through a three-step process including variance thresholding, K-best univariate selection, and LASSO regression, followed by inputting them into various ML models. The XGBoost classifier performed best in the validation cohort and was chosen as the optimal classifier. A probable explanation for this outcome is that the XGBoost algorithm achieves a delicate balance between model complication and learning capability through its unique gradient-boosting mechanism and regularization strategies. This balance allows XGBoost to achieve superior generalization performance with limited sample data, thereby maintaining high predictive accuracy when faced with new, unseen data (25). Li et al. (26) developed an ML model based on the ACS NSQIP database to predict major adverse cardiovascular events within 30 days after CAS. Our research included 2,093 patients from 2011 to 2021, with 6.2% experiencing MACE. The results also confirmed that the XGBoost model did best, realizing 0.93 AUC.

Our final model retained nine key radiomics features, which were broadly categorized into three groups: first-order histogram features, textural features, and wavelet features. First-order histogram features quantify the peakedness and dispersion of PVAT attenuation. Greater dispersion indicates the concurrent presence of low-attenuation lipid-rich regions and areas with relatively higher attenuation, such as early fibrotic tissue or small calcific deposits. This heterogeneous attenuation pattern reflects inflammatory remodeling of PVAT, characterized by a shift from predominantly lipid-rich tissue to areas with increased fibro-inflammatory components (27). The pattern of PVAT inflammation can promote a more unstable plaque phenotype, making the plaque surface more susceptible to disruption, thereby increasing stroke risk in ACS patients (28). Textural features assess the similarity of neighboring voxels and the size distribution of high- and low-attenuation clusters: large low-attenuation clusters suggest greater lipid content, whereas large high-attenuation clusters may represent compensatory fibrotic or calcified changes; the coexistence of these signals is often associated with increased risk of unstable plaque (29). Because plaque instability is a major pathway leading to ischemic stroke in carotid atherosclerosis, the heterogeneous texture patterns captured by radiomics likely reflect an inflammatory microenvironment associated with a higher risk of stroke in patients with asymptomatic carotid stenosis. Wavelet-based radiomics features provide multiscale analysis to reveal subtle heterogeneity within PVAT. High-frequency components reflect localized pathologies such as microcalcifications or fibrosis, whereas low-frequency features characterize the overall lipid distribution patterns (30). These multiscale patterns are believed to capture subclinical changes related to plaque destabilization, including surface irregularity, intraplaque hemorrhage, or microcalcification (31). Such pathological alterations have been associated with an increased risk of future ischemic stroke. Collectively, these radiomics features not only depict PVAT inflammatory activity but also map upstream biological processes that promote plaque vulnerability and ultimately lead to ischemic stroke (13). This mechanistic linkage helps explain why PVAT radiomics demonstrated strong predictive power in our model.

In the validation cohort, the combined model and radiomics model did not significantly differ in terms of the AUC, DCA, NRI, or IDI. These findings reinforce the constraints of clinical characteristics while highlighting the distinctive value of radiomics markers for stroke prediction in ACS patients. Radiomics employs advanced statistical techniques to assess the distribution and variability of pixel intensities within images (32). Texture features reveal the relationships between adjacent pixels, providing insights into subtle pathological changes within this heterogeneity. This approach facilitates the comprehensive quantitative analysis of CTA imaging data, providing clinically actionable information to support medical decision-making.

This research had limitations. First, its retrospective nature inherently introduces biases, and the sample size was limited. Future prospective studies and larger datasets from multiple centers are needed to validate our predictive model. Second, this study did not include pathological validation to directly confirm the association between PVAT radiomics features and inflammation. Pathological correlation will be an important focus of future research to deepen the mechanistic understanding of PVAT-based radiomics. Lastly, inflammation evolution is dynamic, and the radiomics features of PVAT may change with disease progression. Single-time point imaging may not fully capture these changes. Future studies could consider investigating the dynamic radiomics of PVAT.

## Conclusion

To sum up, we constructed and verified a combined model and corresponding nomogram that incorporates clinical features, conventional CTA features, and PVAT radiomics features to forecast short-term stroke risk in ACS patients. Risk calculated by nomogram aids in identifying individuals at high-risk for stroke among ACS patients, thereby identifying those who may benefit from surgical intervention. Consequently, this predictive model offers crucial supplementary information for prognosis assessment and guiding treatment decisions, ultimately reducing the incidence of ischemic stroke. The model can be integrated into workstations as a component of existing AI-assisted vascular imaging software, providing decision support for radiologists and vascular surgeons to enhance clinical application. Future studies with extended follow-up are warranted to determine whether the model also retains predictive value for long-term stroke risk.

## Data Availability

The raw data supporting the conclusions of this article will be made available by the authors, without undue reservation.
