# Supplementary material for: Development of a machine learning-based radiomics model of perivascular adipose tissue for predicting stroke risk in patients with asymptomatic carotid stenosis: a multicenter study
Source: Front Radiol. 2026 Jan 21;5:1738298. doi: 10.3389/fradi.2025.1738298 (PMC12868272; doi:10.3389/fradi.2025.1738298)
Supplement: Supplementary file 1 [file Datasheet1.docx]

**Supplementary Materials**

**1.Supplementary Methods**

**Appendix E1.The definition of stroke during follow-up**

**Appendix E2.Image preprocessing**

**Appendix E3.Repeatability analysis**

**Appendix E4.Methods of Z-score normalization**

**Appendix E5.The three-step procedure of feature selection**

**Appendix E6.The rationales and considerations behind the choice of the five machine learning (ML) classifiers**

**Appendix E7.The optimal hyperparameter combinationsfor each machine learning classifier**

1. **Supplementary Tables**

**Table S1.** The parameters of different CTA devices

**Table S2.** Comparison of Clinical Characteristics and CTA Features between Stroke and Non-stroke Groups in Patients with ACS in the training, internal validation cohort,external validation cohort1 and external validation cohort2

**Table S3.** Univariate and multivariate logistic regression analyses were used to screen for risk factors associated with stroke in ACS patients in the training cohort.

**3.Supplementary Figures**

**Figure S1.** (a) Feature coefficients corresponding to the value of parameter λ. Each curve represents the change trajectory of each independent variable. (b) The most valuable features were screened out by tuning λ using LASSO via minimum binomial deviation. As the parameter λ increases, the binominal deviance decreases gradually to the lowest point. The dotted vertical line represents the optimal log (λ) value. (c) The selected 9 radiomics features from CTA images with the most discriminative value.

**Figure S2.** The correlation heatmap of the selected radiomics features from CTA.

**Figure S3.** ROC analysis results (a) and DeLong’s tests (P value) of diferent radiomics signatures (b) in the internal validation cohort (left)、external validation cohort 1 （middle）and external validation cohort 2 (right). ACC, accuracy; AUC, area under the curve; KNN, k-nearest neighbors; SVM, support vector machine; LR, logistic regression; BernoulliNB, BernoulliNB naive Bayes;XGBoost, eXtreme Gradient Boosting ROC, receiver operating characteristic; SEN, sensitivity; SPE, specifcity;

**Figure S4.** Distribution of Rad-scores based on the XGBoost classifier between the ACS patients between non-stroke and stroke groups in the (a) training, (b) internal validation cohort, (c)external validation cohort 1and (d) external validation cohort2.

**Figure S5.** Confusion matrices for different models in classifying whether ACS patients had a stroke in the training set(a), internal validation set(b), external validation set 1(c), and external validation set 2(d).Confusion matrices of the clinical model, radiomics model, and fusion model. The color depends on the number inside the square: the higher the number, the darker the color.

**Figure S6.** The performance of different models in predicting the stroke risk level of ACS patients was assessed using the Net Reclassification Improvement (NRI) and the Integrated Discrimination Improvement (IDI).

FiguresS6 a to d represent the NRI for the training set, internal validation set, external validation set 1, and external validation set 2, respectively.

FiguresS6 e to h represent the IDI for the training set, internal validation set, external validation set 1, and external validation set 2, respectively.

The corresponding significance is shown by the color of the blocks (blue indicates P≥0.05, and red indicates P<0.05).

**Figure S7.**Visualization of PVAT radiomic features derived in patients with and without stroke. (A, B) CTA images of a patient with stroke. (F, G) CTA images of a patient without stroke, with PVAT regions outlined by blue circles. (C–E, H–J) Pseudo-color maps of the top three radiomic features.

**Appendix E1**

**The definition of stroke during follow-up.**

Stroke events during follow‑up were defined as (i) an acute focal neurological deficit persisting ≥24 h, and (ii) a corresponding new ischaemic lesion on diffusion‑weighted MRI or, when MRI was unavailable, on non‑contrast CT. Only ischaemic strokes were counted; minor stroke,intracerebral haemorrhage, subarachnoid haemorrhage, and transient ischaemic attack were excluded.

**Appendix E2**

**Image preprocessing**

To mitigate image heterogeneity, all CTA images were resampled to a uniform voxel size (1 mm × 1 mm × 1 mm) to reduce variability in radiomic feature values caused by differing voxel sizes. Furthermore, Hounsfield units (HU) were standardized using a fixed histogram bin width of 25 HU. Additionally, images were smoothed using a Gaussian kernel with a standard deviation of 0.8 mm to reduce noise.

**Appendix E3**

**Repeatability analysis**

To ensure the reproducibility of the extracted features, one radiologist (reader A, with 10 years of experience) performed a second delineation of volumes of interest (VOIs) from 30% of randomly selected images (175 samples) 2 weeks later to evaluate the intra-observer repeatability. Then, another radiologist (reader B, with 10 years of experience) independently drew the VOIs from selected images to assess the inter-observer repeatability. The intra- and inter-observer repeatability were measured by the intraclass correlation coefficients (ICCs). Features with ICCs greater than 0.8 were considered to have good reproducibility and were included in subsequent analyses.

**Appendix E4**

**Methods of Z-score normalization**

The standardized formula is as follows:

$$f\left( x \right)=\frac{s(x-\mu_{x}）}{\delta_{x}}$$

where $x$ is the original intensity, $f\left( x \right)$ is the normalized intensity, $\mu$ and $\delta$ are the mean and variance, respectively, and $s$ is an optional scaling factor (set to 1 by defaul

**Appendix E5**

**The three-step procedure of feature selection**

To reduce data dimensionality and mitigate overfitting, a systematic three-step feature selection procedure was applied prior to model development.

(1) Variance Thresholding: Features with low variance (threshold = 0.2) were excluded to remove non-informative variables. Additionally, constant or near-constant features—often arising from intrinsic data characteristics or discretization—were removed to minimize potential technical artefacts.

(2) Univariate K-Best Selection: The 100 highest-ranking features were then retained based on univariate statistical association with the target variable, ensuring that the most relevant predictors were preserved.

(3) LASSO Regression: Finally, LASSO regression was used to further refine the selected feature set by reducing multicollinearity and retaining the most informative predictors for the final machine learning model.10-fold cross-validation was used to select the optimal λ. And the λ yielding the minimum mean cross-validated deviance was chosen for the final model.

**Appendix E6**

**The rationales and considerations behind the choice of the five machine learning (ML) classifiers**

The k-Nearest Neighbors (KNN) algorithm classifies based on the similarity of a data point's features to points within the dataset, considering its "k" nearest neighbors. It was selected for its simplicity and interpretability in classification tasks. The Support Vector Machine (SVM) algorithm, applicable for linear or non-linear classification, aims to identify the optimal separating hyperplane, maximizing the margin between the hyperplane and the nearest data points. Its effectiveness in high-dimensional spaces makes it suitable for handling the complex feature sets typical in radiomics data. Logistic Regression (LR), a widely used binary linear classifier, employs a sigmoid function for non-linear transformation and learns the posterior probability of a single sample using a log-likelihood estimation function. It was included for its proficiency in providing probabilistic outputs and its interpretability in clinical settings. The Bernoulli Naive Bayes (BernoulliNB) algorithm is based on the application of Bayes' theorem, assuming conditional independence between each pair of features. It is particularly effective for categorical data and is commonly used in classification tasks. The XGBoost (Extreme Gradient Boosting) algorithm operates by constructing a series of decision trees, where each subsequent tree corrects the errors of the preceding ones. It was selected for its efficiency, flexibility, and ability to handle various data types and tasks. Each classifier was chosen for its unique approach to learning and pattern recognition, ensuring that the models can capture various aspects of the data. Furthermore, these classifiers are commonly used in previous ML-related studies. Including diverse ML classifiers allows us to compare and construct the optimal predictive model for our specific application.

**Appendix E7**

**The optimal hyperparameter combinationsfor each machine learning classifier**

The optimal hyperparameter combinations for the five classifiers are as follows:

(1)KNN: n_neighbors=10;weights=uniform

(2)SVM: C=1, kernel=‘rbf’;

(3)LR: penalty=‘l2’, C=1, solver=‘liblinear’, class_weight =‘None’;

(4)BernoulliNB: alpha=1.0，binarize=0.5，fit_prior=True，class_prior=None

(5)XGBoost:learning_rate=0.1, max_depth= 2.

| **Table S1.** The parameters of different CTA devices | | | | | | | | |
| --- | --- | --- | --- | --- | --- | --- | --- | --- |
|  | tube voltage | tube current | pitch | matrix | collimation width | slice thickness | interval | gantry rotation time |
| **Center1** | **100kV** | **140mAs** | **1.0** | **512×512** | **128×0.6mm** | **1mm** | **0.7mm** | **0.25s** |
| **Center2** | **100kV** | **140mAs** | **1.0** | **512×512** | **128×0.6mm** | **1mm** | **0.7mm** | **0.25s** |
| **Center3** | **120kV** | **150mAs** | **0.992** | **512×512** | **64×0.6mm** | **0.625mm** | **0.625mm** | **0.5s** |

| **Table S2.** Comparison of Clinical Characteristics and CTA Features between Stroke and Non-stroke Groups in Patients with ACS in the training, internal validation cohort,external validation cohort1 and external validation cohort2 | | | | | |
| --- | --- | --- | --- | --- | --- |
| **Characteristic** | **Training cohort**  **(n =188)** | **Internal validation cohort**  **(n = 85)** | **External validation cohort1**  **(n = 157)** | **External validation cohort2**  **(n = 152)** | **P** |
| Age, years* | 56.48 ± 10.60 | 55.80 ± 10.20 | 57.79 ± 10.32 | 57.87 ± 12.44 | 0.369 |
| Sex(%) |  |  |  |  | 0.887 |
| Female | 96 (51.06) | 44 (51.76) | 77 (49.04) | 72 (47.37) |  |
| Male | 92 (48.94) | 41 (48.24) | 80 (50.96) | 80 (52.63) |  |
| BMI* | 24.46 ± 1.00 | 24.55 ± 0.93 | 24.89 ± 2.43 | 24.46 ± 1.00 | 0.298 |
| Smoke status(%) |  |  |  |  | 0.550 |
| NO | 93 (49.47) | 49 (57.65) | 87 (55.41) | 79 (51.97) |  |
| YES | 95 (50.53) | 36 (42.35) | 70 (44.59) | 73 (48.03) |  |
| Diabetes(%) |  |  |  |  | 0.756 |
| NO | 85 (45.21) | 42 (49.41) | 73 (46.50) | 77 (50.66) |  |
| YES | 103 (54.79) | 43 (50.59) | 84 (53.50) | 75 (49.34) |  |
| Hypertension(%) |  |  |  |  | 0.504 |
| NO | 87 (46.28) | 45 (52.94) | 67 (42.68) | 71 (46.71) |  |
| YES | 101 (53.72) | 40 (47.06) | 90 (57.32) | 81 (53.29) |  |
| Hyperlipidemia(%) |  |  |  |  | 0.658 |
| NO | 86 (45.74) | 42 (49.41) | 77 (49.04) | 80 (52.63) |  |
| YES | 102 (54.26) | 43 (50.59) | 80 (50.96) | 72 (47.37) |  |
| CAD(%) |  |  |  |  | 0.355 |
| NO | 88 (46.81) | 48 (56.47) | 77 (49.04) | 68 (44.74) |  |
| YES | 100 (53.19) | 37 (43.53) | 80 (50.96) | 84 (55.26) |  |
| Family history(%) |  |  |  |  | 0.727 |
| NO | 93 (49.47) | 43 (50.59) | 81 (51.59) | 69 (45.39) |  |
| YES | 95 (50.53) | 42 (49.41) | 76 (48.41) | 83 (54.61) |  |
| Soft plaque(%) |  |  |  |  | 0.773 |
| NO | 82 (43.62) | 42 (49.41) | 71 (45.22) | 73 (48.03) |  |
| YES | 106 (56.38) | 43 (50.59) | 86 (54.78) | 79 (51.97) |  |
| Ulcerated plaque(%) |  |  |  |  | 0.056 |
| NO | 135 (71.81) | 61 (71.76) | 73 (46.50) | 72 (47.37) |  |
| YES | 53 (28.19) | 24 (28.24) | 84 (53.50) | 80 (52.63) |  |
| Plaque thickness(mm)* | 4.23 ± 1.32 | 4.22 ± 1.33 | 4.05 ± 1.18 | 4.10 ± 1.24 | 0.514 |
| Plaque length(mm)* | 14.93 ± 5.94 | 15.44 ± 6.45 | 15.70 ± 5.48 | 15.91 ± 5.54 | 0.433 |
| TC(mmol/L) | 3.51 (3.29,3.69) | 3.49 (3.31,3.64) | 3.47 (3.34,3.60) | 3.45 (3.20,3.66) | 0.401 |
| TG(mmol/L) | 1.19 (1.04,1.32) | 1.12 (1.01,1.26) | 1.13 (0.99,1.26) | 1.18 (1.02,1.32) | 0.075 |
| HDL-C(mmol/L) | 1.02 (0.93,1.11) | 1.02 (0.94,1.12) | 1.00 (0.92,1.10) | 1.00 (0.94,1.08) | 0.280 |
| LDL-C(mmol/L) | 1.86 (1.59,2.04) | 1.90 (1.56,2.10) | 1.91 (1.75,2.05) | 1.88 (1.72,2.09) | 0.072 |
| Abbreviations:ACS,asymptomatic carotid stenosis;CTA,computed tomography angiography;BMI, body mass index; CAD, coronary artery disease; TC, total cholesterol; TG, triglyceride; HDL-C, high-density lipoprotein cholesterol; LDL-C, low-density lipoprotein cholesterol | | | | | |

| **Table S3.** Univariate and multivariate logistic regression analyses were used to screen for risk factors associated with stroke in ACS patients in the training cohort. | | | | |
| --- | --- | --- | --- | --- |
| **Variable** | **Univariate analysis** | | **Multivariate analysis** | |
|  | **OR (95% CI)** | ***P* value** | **OR (95% CI)** | ***P* value***** |
| Age (years) | 1.00 (0.98-1.03) | 0.819 |  |  |
| Sex | 1.62 (0.89-2.96) | 0.114 |  |  |
| BMI | 1.05 (0.78-1.42) | 0.734 |  |  |
| Smoke status | 0.65 (0.36-1.18) | 0.157 |  |  |
| Diabetes | 0.85 (0.47-1.54) | 0.584 |  |  |
| Hypertension | 3.91(2.07-7.63) | **<0.001** | 3.86 (1.97-7.82) | **<0.001** |
| Hyperlipidemia | 0.55 (0.30-1.00) | 0.052 |  |  |
| CAD | 0.59 (0.32-1.07) | 0.085 |  |  |
| Family history | 1.01 (0.56-1.83) | 0.968 |  |  |
| Soft plaque | 4.64 (2.40-9.37) | **<0.001** | 4.29 (2.03-9.43) | **<0.001** |
| Ulcerated plaque | 2.30 (1.20-4.43) | **0.012** | 1.20 (0.56-2.57) | 0.637 |
| Plaque thickness | 1.00 (0.80-1.26) | 0.968 |  |  |
| Plaque length | 0.99 (0.94-1.04) | 0.681 |  |  |
| TC | 1.32 (0.49-3.60) | 0.583 |  |  |
| TG | 0.80 (0.19-3.34) | 0.760 |  |  |
| HDL | 0.40 (0.07-2.12) | 0.297 |  |  |
| LDL | 0.96 (0.45-2.05) | 0.911 |  |  |
| Note: *Those variables found significant at *P* < 0.05 in univariable analyses were entered into multivariable analyses.  Abbreviations:ACS,asymptomatic carotid stenosis; BMI, body mass index; CAD, coronary artery disease; TC, total cholesterol; TG, triglyceride; HDL-C, high-density lipoprotein cholesterol; LDL-C, low-density lipoprotein cholesterolOR, odds ratio; CI, confidence interval. | | | | |

**Figure S1.** (a) Feature coefficients corresponding to the value of parameter λ. Each curve represents the change trajectory of each independent variable. (b) The most valuable features were screened out by tuning λ using LASSO via minimum binomial deviation. As the parameter λ increases, the binominal deviance decreases gradually to the lowest point. The dotted vertical line represents the optimal log (λ) value. (c) The selected 9 radiomics features from CTA images with the most discriminative value.


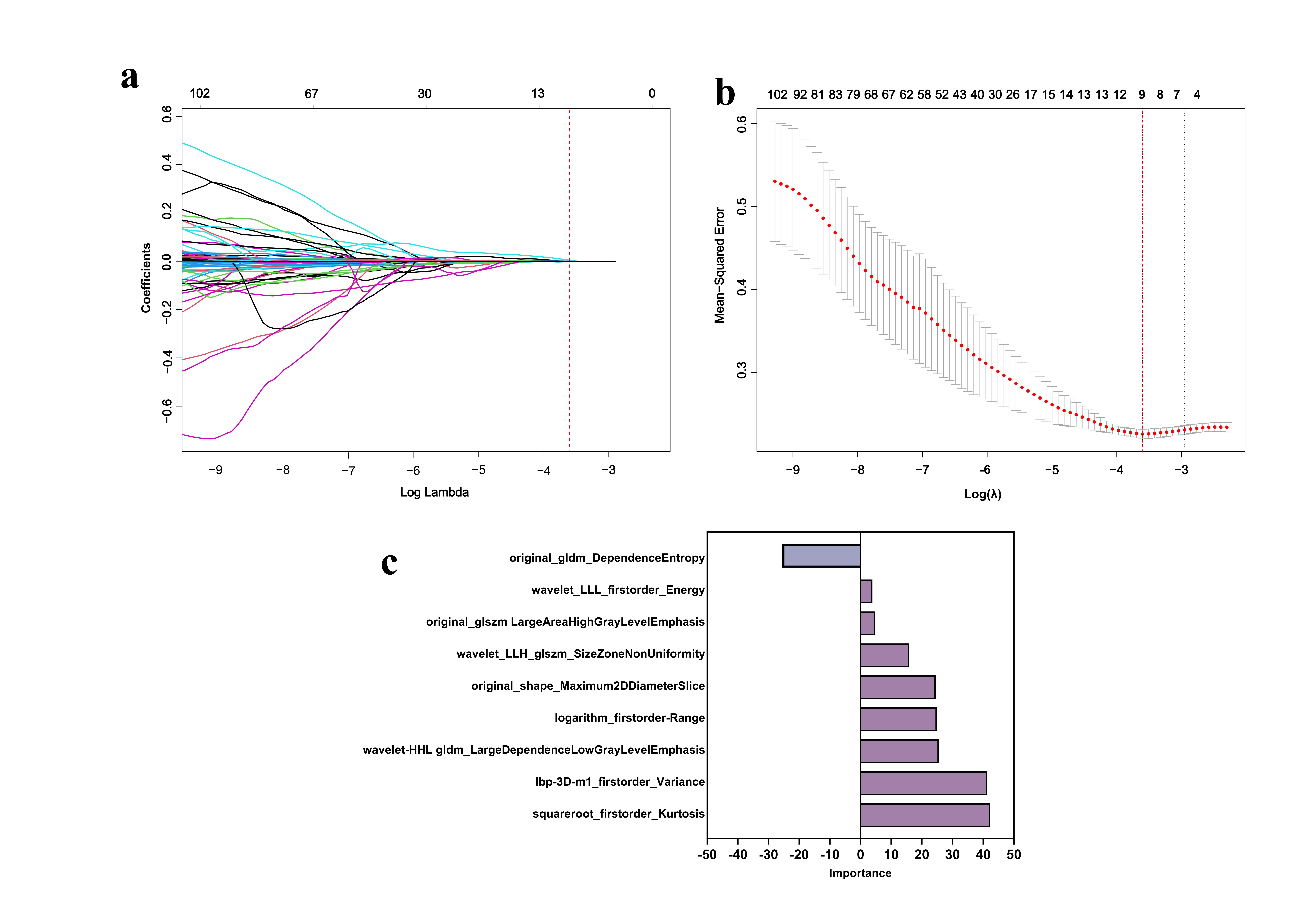


**Figure S2.** The correlation heatmap of the selected radiomics features from CTA


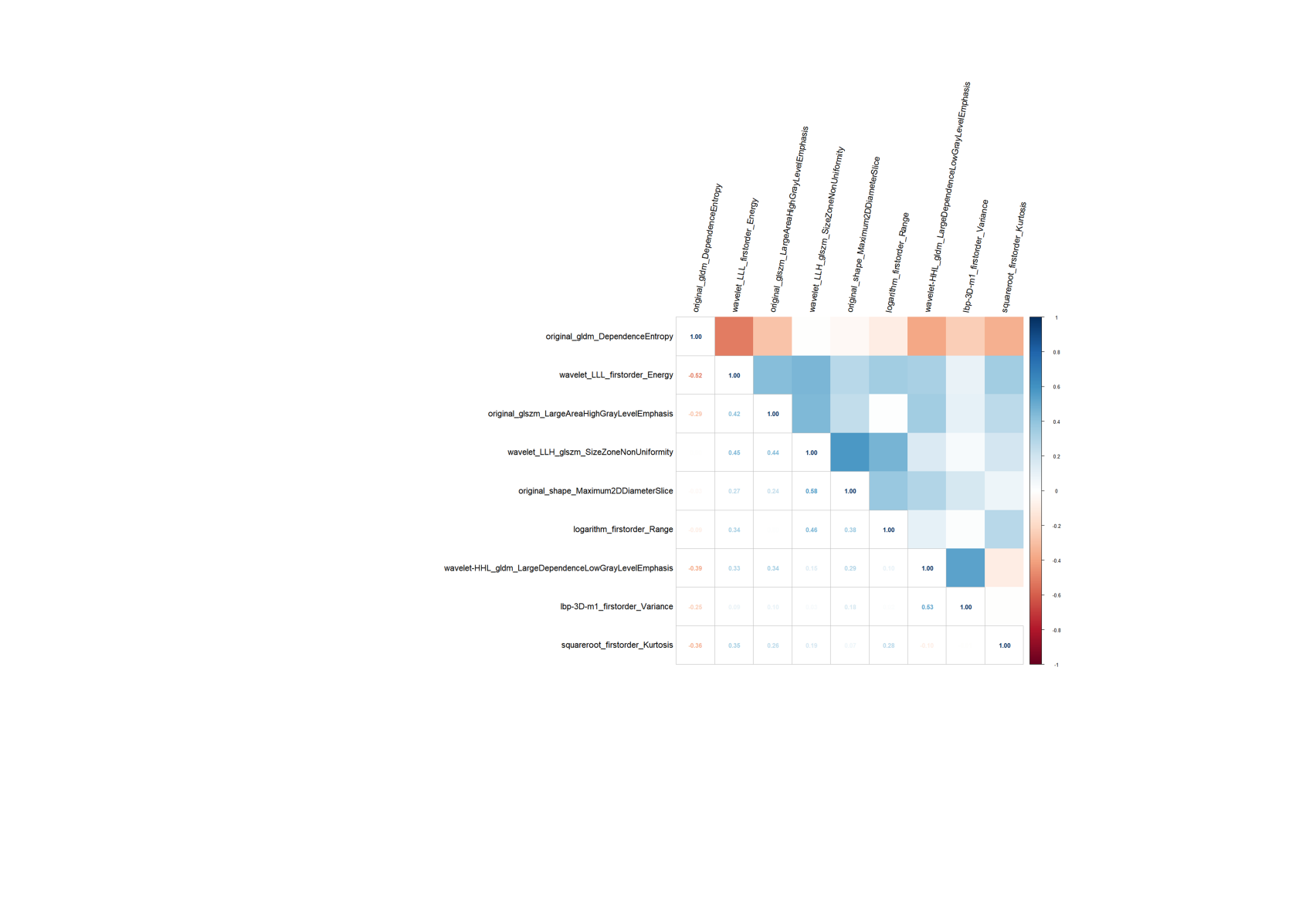


**Figure S3.** ROC analysis results (a) and DeLong’s tests (P value) of diferent radiomics signatures (b) in the internal validation cohort (left)、external validation cohort 1 （middle）and external validation cohort 2 (right). ACC, accuracy; AUC, area under the curve; KNN, k-nearest neighbors; SVM, support vector machine; LR, logistic regression; BernoulliNB, BernoulliNB naive Bayes;XGBoost, eXtreme Gradient Boosting ROC, receiver operating characteristic; SEN, sensitivity; SPE, specifcity;


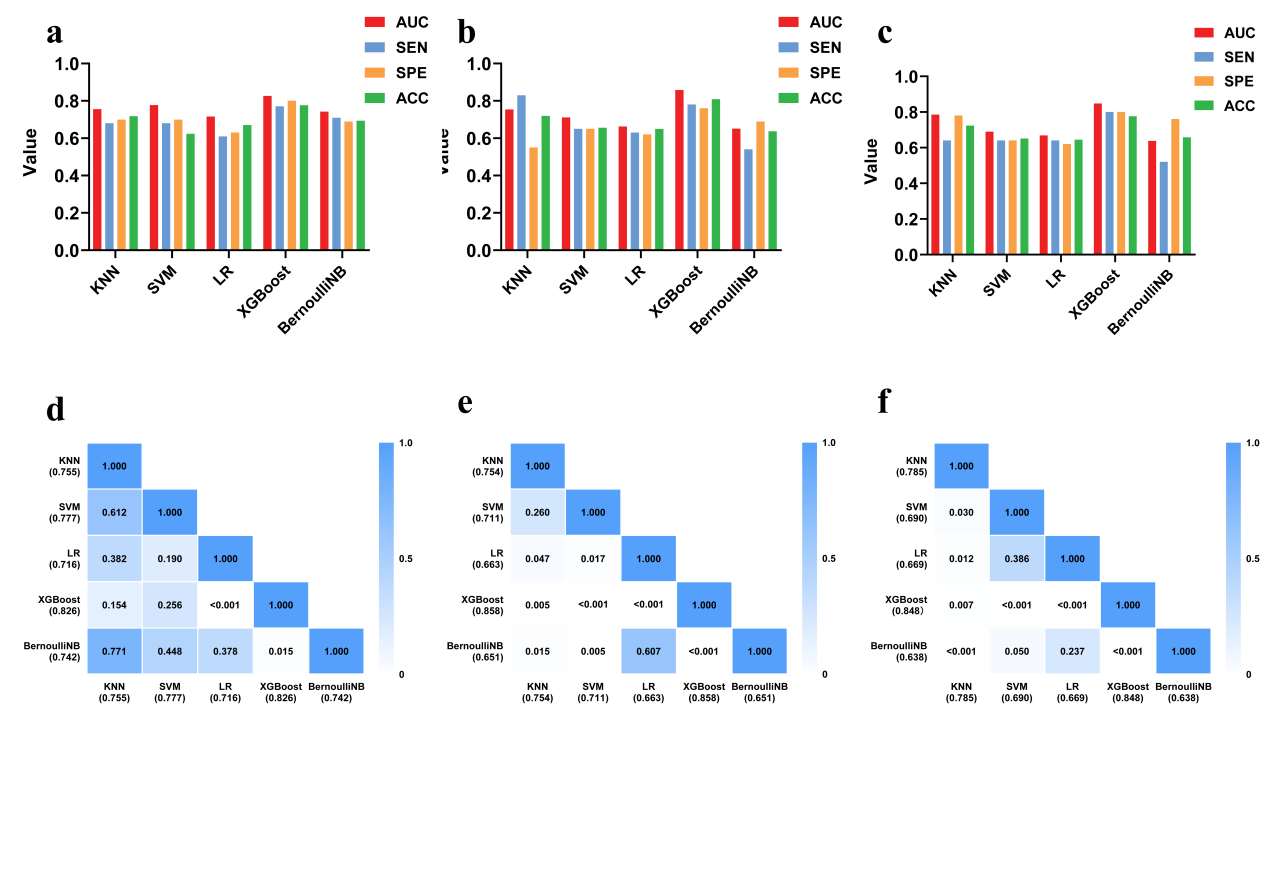


**Figure S4.** Distribution of Rad-scores based on the XGBoost classifier between the ACS patients between non-stroke and stroke groups in the (a) training, (b) internal validation cohort, (c)external validation cohort 1and (d) external validation cohort2.


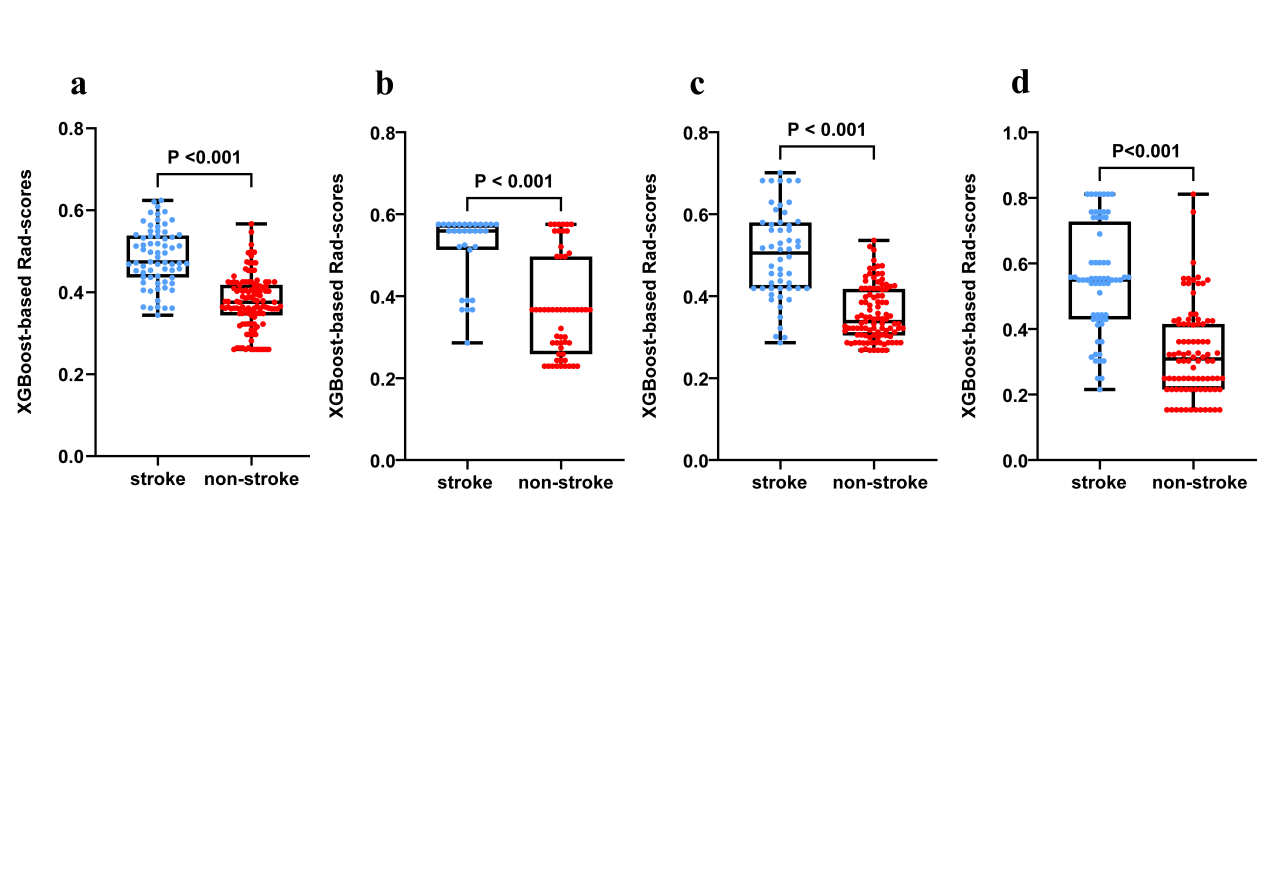


**Figure S5.** Confusion matrices for different models in classifying whether ACS patients had a stroke in the training set(a), internal validation set(b), external validation set 1(c), and external validation set 2(d).Confusion matrices of the clinical model, radiomics model, and fusion model. The color depends on the number inside the square: the higher the number, the darker the color.


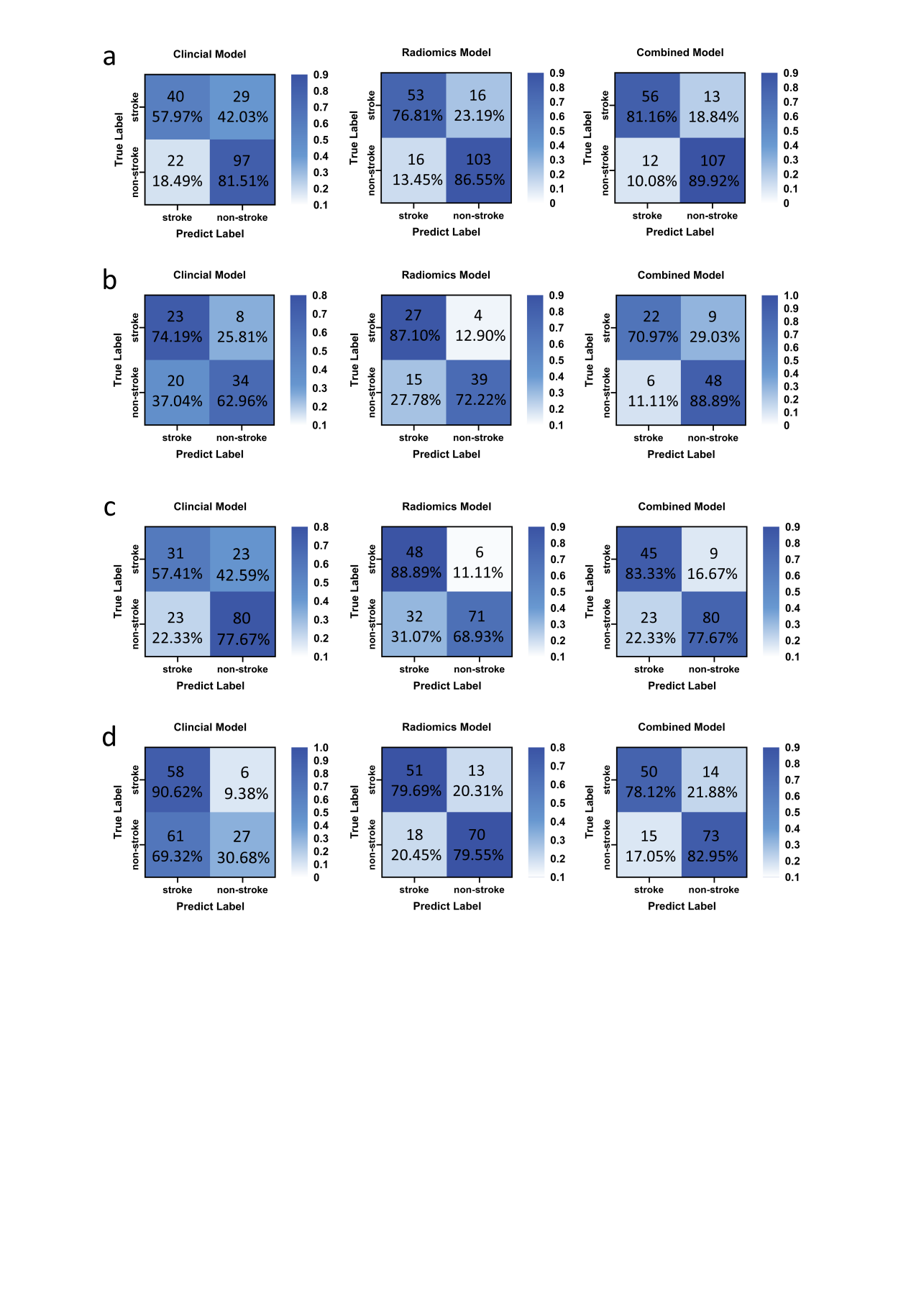


**Figure S6.** The performance of different models in predicting the stroke risk level of ACS patients was assessed using the Net Reclassification Improvement (NRI) and the Integrated Discrimination Improvement (IDI).

FiguresS5 a to d represent the NRI for the training set, internal validation set, external validation set 1, and external validation set 2, respectively.

FiguresS5 e to h represent the NRI for the training set, internal validation set, external validation set 1, and external validation set 2, respectively.

The corresponding significance is shown by the color of the blocks (blue indicates P≥0.05, and red indicates P<0.05).


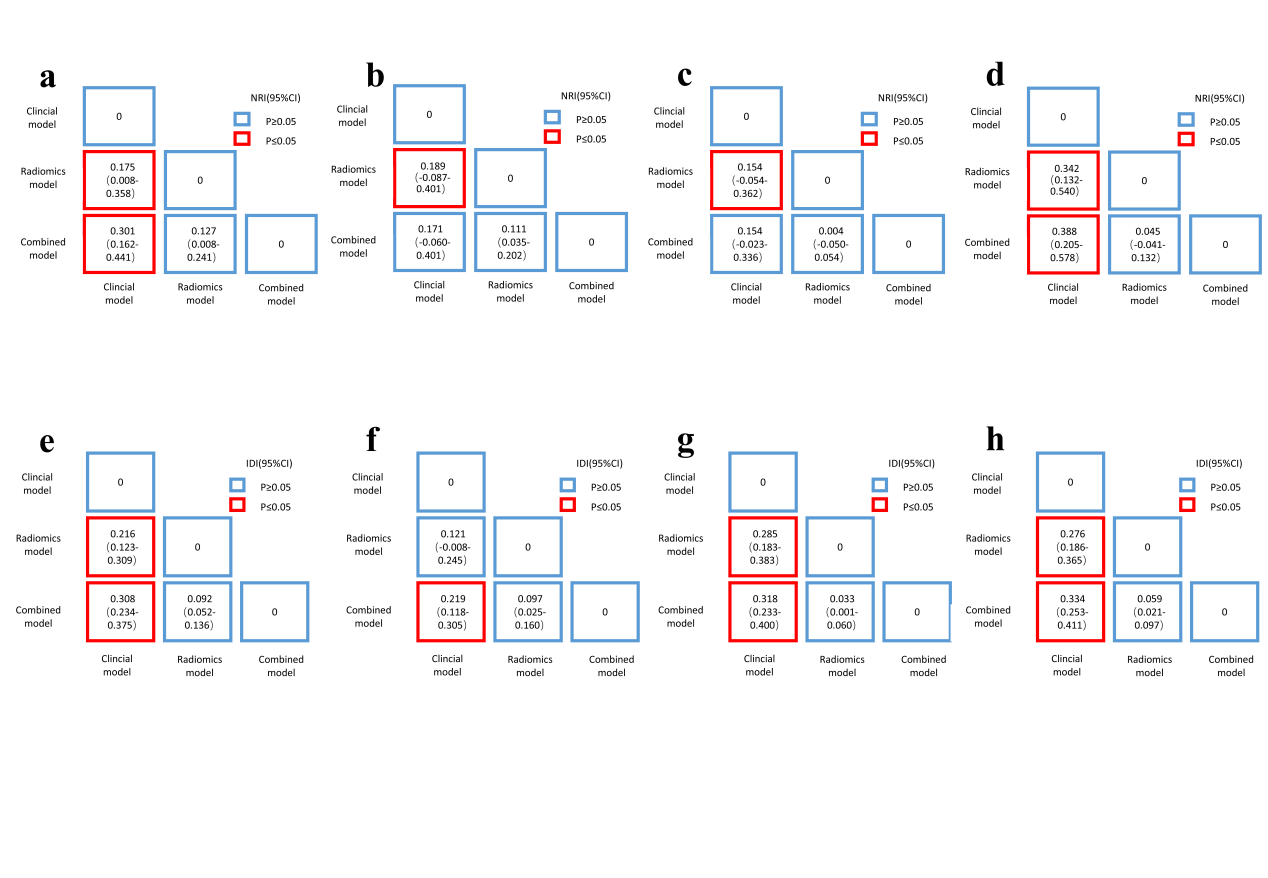


**Figure S7.**Visualization of PVAT radiomic features derived in patients with and without stroke. (A, B) CTA images of a patient with stroke. (F, G) CTA images of a patient without stroke, with PVAT regions outlined by blue circles. (C–E, H–J) Pseudo-color maps of the top three radiomic feature.
